# Association between trail use and self-rated wellness and health

**DOI:** 10.1186/s12889-020-8273-0

**Published:** 2020-01-30

**Authors:** Abbas Smiley, William D. Ramos, Layne M. Elliott, Stephen A. Wolter

**Affiliations:** 1 Department of Surgery, Westchester Medical Center, School of Medicine, New York Medical College, Valhalla, New York, NY 10595 USA; 2 Recreation, Park, and Tourism Studies Department, Indiana University School of Public Health- Bloomington, Bloomington, Indiana USA; 3 Eppley Institute for Parks and Public Lands, Indiana University School of Public Health- Bloomington, Bloomington, Indiana USA

**Keywords:** Trails, Built-environment, Public policy, Health, Wellness, Physical activity

## Abstract

**Background:**

Incorporating trail use into daily activity routines could be an important venue to increase a population’s physical activity. This study presents important health impacts of trail use.

**Methods:**

A cross-sectional study was conducted on 8 trails throughout the State of Indiana. A mix of urban, suburban, and rural trails were selected. Recruitment sessions were completed during four 1-week periods throughout the study in various locations and at various times of day on each trail between April and October 2017. Data were collected through online and paper surveys. For each type of physical activity, a generalized additive model for self-rated wellness and health was built adjusting for demographics, socioeconomic status, amounts of physical activity on trails, mood status, sleep pattern, diet and smoking habit. The plots of estimated smoothing spline function with 95% confidence band were pictured. All statistical analyses were conducted using R.

**Results:**

The final sample size included 1299 trail users; 92% were White, 79% aged 18–65 years, 71% were married and 56% were male. Biking, walking and running were the main activities with 52, 29 and 19%, respectively. Female to male ratio was 3:2 in walkers vs. 2:3 in runners and bikers. Runners were significantly younger than the other two groups. Runners also had the highest percentage of college graduates and above, the highest rate of employment, the highest income, and the lowest percentage of being retired among the three groups. They more commonly used the trails alone than the walkers and bikers. Bikers had the highest rate of job satisfaction. They also showed a better mean score of mood than that the walkers and runners. There was a linear association between walking and self-rated wellness and health, and a curved association between running/biking and self-rated wellness and health. Running < 6.5 miles/week and biking > 14 miles/week were associated with steeper rise in self-rated wellness and health.

**Conclusions:**

Employed educated married middle-aged people had the highest prevalence of walking, running or biking. The higher the walking, the higher self-rated wellness and health. A similar association was observed for running up to 6.5 miles/week or biking > 14 miles/week.

## Background

Bicycle and pedestrian trails are frequently constructed in the U.S. for varying reasons including as alternate transportation, for economic development, and for health promotion. Incorporating bicycling, walking and running into daily physical activities depends on many elements including perceived environmental factors [1–3]. In a quasi-experimental study [4] the investigators compared the amount of physical activity of people around a new built trail in Tennessee with that in two control neighborhoods that lacked any trail. They found a significant increase in walking and biking in trail neighborhood compared to the control areas. Also, a pre- and post-campaign of a new trail launch in Australia on 450 adults showed significant increase in biking time and biking count in the monitored areas [5]. Those living near a walking/biking trail were more likely to walk 150 min/week compared to those not living close to a walking/biking trail. This was shown by a multivariable logistic regression model adjusted for 12 independent variables in a cross-sectional survey conducted on 1211 people in Texas [6]. People who started to use the trail have reported an increase in their amount of walking since they began using the trails [7]. These findings mean greater levels of physical activity are expected by preparing the pedestrian connectivity of the built environment. In addition, several studies have shown positive effects of recreational cycling and walking on health outcomes [8–10] and reduction of all-cause mortality rate [11].

The State of Indiana, which ranks 39th out of 50 U.S. States in overall health [12] has a policy to construct bicycle pedestrian trails within 5 miles of all Indiana residents by the year 2020. As of 2017, that goal was 94.4% achieved [13]. As a public policy, the health outcomes are not specified in the creation of trails proximate to Indiana residents. Measuring health outcomes of trail users is an important measure of the implementation and effectiveness of Indiana trail policy. Design attributes from the 2001 Indiana Trail Study [14] served to provide methodological guidance for the 2017 Indiana Trails Study [15]. This study outlines important health outcomes and data related to the Indiana policy to construct bicycle and pedestrian trails throughout the State, as well as analysis of the health impacts of trail use. The primary goal was to picture the overall characteristics of population who use the trails and the patterns of their physical activities. The secondary goal was to assess the association of various trail physical activities with self-rated wellness and health index.

## Methods

The 2017 Indiana Trails Study was a cross-sectional study conducted on 8 trails throughout the State of Indiana. This study was suggested in part by Indiana trail advocates to replicate a previous study conducted in 2001. Specifically, the 2017 study’s methods included data points from the following:

- Traffic (user) counts collected via trail counters at select trail segments
- Online surveys (or paper-mail surveys upon request)

The Office of Research Compliance at Indiana University approved the study protocol.

### Population/study sites

Participating trails were selected from their ability and willingness to fully participate based on the managing agency staff, volunteer network, and available equipment (trail counters). Trails were also selected to create a mix of urban, suburban, and rural trails from all regions of the state in order to gather the most diverse and representative data set possible. No single definition of “urban”, “suburban”, or**”** rural” fit the needs of this study so a compilation of sources including the Indiana Department of Transportation, the U.S. Census Bureau, and other anecdotal sources were used to create the following definitions: Urban defined as areas of dense residential, commercial, or industrial. It includes medium to larger city centers. Suburban defined as areas of average density of single-family homes and light retail commercial. Rural defines as residential or agricultural areas of at least one acre on average, farmland, or open range or forest.

In order to recruit survey participants, trained volunteers from the trail management agencies were stationed at specified trailheads at researcher specified times and days to distribute study information including the link to the online trail survey. Recruitment sessions were completed during four 1-week periods throughout the study in various locations and at various times of day on each trail between April and October.

Data collection weeks were April 10–16, June 5–11, August 6–12 and October 2–8. Data collection times for trail users were defined as early morning (6–8 am), mid-morning (8–11 am), midday (11 am - 2 pm), afternoon (2–5 pm), and evening (5–8 pm or until dusk, if before 8 pm). Volunteers were scheduled to recruit survey participants during these time frames. This scheduling of the survey recruitment effort is similar to scheduling from the 2001 study. Factors considered when selecting trailheads included location and survey number. Popular trailheads were selected in order to intercept users when starting or ending trail use. The target number of trail user survey responses relied on the populations of participating communities. Survey participants were directed to take on online survey with paper versions of both the trail user and non-user surveys available upon request.

### Data gathering/instrumentation

Demographic information, socioeconomic status, physical activity levels, mood status, smoking, sleep, and diet data were collected through the online and paper surveys. The Recreation Trail Evaluation Survey (RTES) was used to gather trail user information about patterns of physical activity and trail use [16]. To evaluate mood, five principal elements of mood were assessed according to the Gallup Well-being Index [17]. Subjects were asked, how many days per week did they experience each of the following symptoms: (a) no energy to get things done, (b) sadness, (c) anger, (d) physical pain, and (e) worry. The sum of the five scores was considered as the overall mood score (0–35) for regression analyses with a higher score indicating a worse mood status. For the sleep assessment, a Mini-Sleep Questionnaire [18] was used. Participants were asked how many days per week they experienced the followings: (a) difficulty falling asleep, (b) waking up too early, (c) use of hypnotic medications, (d) falling asleep during the day, (e) feeling tired upon waking up in the morning, (f) snoring, (g) experiencing mid-sleep awakenings, (h) experiencing headache on awakening, (i) excessive daytime sleepiness, and (j) excessive movement during sleep. The sum of the 10 scores was considered as sleep score (0–70) for regression analyses. The higher the score, the worse the sleep pattern. To have a rough index of diet, subjects were asked how many days per week they ate fast food and how many days per week they ate less than four/five servings of fruits and vegetables. The sum of the two scores was considered as the diet score (0–14) for regression analyses. Again, the higher the score, the worse the diet pattern. Smoking habits were also inquired. The last question of the survey asked trail users how highly they rated their wellness and health out of 10, when 10 was the best and a score of zero denoted the worst condition.

## Data analysis/calculation

Trail users were categorized into three groups according to their main type of physical activity in trails; i.e., walking, running and biking. Their demographic, socioeconomic status (SES) and trail activity characteristics were compared. For each type of physical activity, a linear regression model for self-rated wellness and health was built adjusting for demographics, SES, the amount of physical activity on trails, mood status, sleep pattern, diet and smoking habit. Similarly, generalized additive models (GAM) were built [19]. GAM is an extension of generalized linear model allowing for nonlinear (smooth) associations between the predictor variables and the outcome. GAM was employed to assess the curved relationship between the independent variable- the amount of physical activity in trails, and the dependent variable- self-rated wellness and health. The assumptions of normality of residuals and the equality of variances in GAM models were checked in order to evaluate the success or failure of the fitting process [19]. If the above-mentioned assumptions were not met, increasing the dimension of the basis (K) in GAM model, considering the square-root or log-transformation of the variable(s) and/or changing the family approach in GAM model were among the solutions. K in GAM model is the choice of dimension of the basis used to represent smooth terms. The actual effective degree of freedom (EDF) is primarily controlled by smoothing the penalty which controls the smoothing curve. The upper limit of EDF is K-1 which is the basis dimension minus one degree of freedom due to identifiability constraint for each smooth term. The choice of basis dimensions amounted to setting maximum possible degrees of freedom. Three GAM models were fitted for self-rated wellness and health according to the amount of the three principal types of physical activity in trails- walking, running or biking. Finally, the plots of estimated smoothing spline function with 95% confidence band were pictured. A two-tailed *p* value less than 0.05 was considered significant. All statistical analyses were conducted using R package software.

## Results

The final sample size included 1299 trail users. One thousand two hundred eleven reported their age of which 962 (79%) aged 18–65 years; 669 of 1204 (56%) who reported their sex were male; 1109 of 1208 (92%) reporting race/ethnic origin were White; 800 of 1208 (66%) reporting employment status were employed/self-employed; 983 of 1118 (88%) reporting income had a household income over $38,000; 854 of 1195 (71%) reporting marital status were married or had a domestic partnership; 917 of 1199 (76%) reporting education level had at least some college education; and 1192 of 1232 (97%) reporting smoking habits were non-smokers.

About 45% stated never eating fast food and 20% specified never eating less than 4–5 servings of fruit/vegetables. 1% stated eating fast food almost every day and 15% specified eating less than 4–5 servings of fruit/vegetables almost every day (6–7 days/week).

The comparison of demographic and SES characteristics of trail users among walkers, runners and bikers is presented in Table 1. Interestingly, the three sets of trail users were significantly different in terms of all presented characteristics in Table 1 except the time of the day for trail use. For instance, female to male ratio was 3:2 in walkers vs. 2:3 in runners and bikers. Runners were significantly younger than the other two groups. Runners also had the highest percentage of college graduates and above, the highest rate of employment, the highest income, and the lowest percentage of being retired among the three groups. They more commonly used the trails alone than the walkers and bikers. Bikers had the highest rate of job satisfaction (Table 1).

**Table 1:** Demographic and SES characteristics of trail users according to their main type of physical activity on trails

Demographic & SES Characteristics	Walkers, %	Runners, %	Bikers, %	*P* value
Age, years				
18–25	3.5	12	4.5	0.0001
26–35	13.5	23	9.5	
36–45	16	32	10	
46–65	45	31	49	
> 65	22	2	27	
Sex				
Female	60	41	36.5	0.0001
Male	40	59	63.5	
Race				
White	90.7	91.6	97	0.0001
Black	3	1	1	
Hispanic	3	7	1.5	
Asian	3	0	0.5	
Indian	0.3	0.4	0	
Marital Status				
Single	19	26	16	0.003
Married	68	67	75	
Widowed	3.5	0	2	
Divorced	9.5	7	7	
Employment				
Homemaker	5	3	3	0.0001
Self-employed	8	10	10	
Student	3	9	2	
Employed	55	75	52	
Retired	28	2	31	
Not Employed	1	1	2	
Job Satisfaction				
< 30%	7	6	4	0.001
30–70%	24	22	19	
> 70%	69	72	77	
Education				
<9th Grade	0.5	0	1.5	0.008
High School	16	11	15	
Technical School	4.5	2	9	
College Graduate	41	45	38	
Graduate School	27.5	28.5	25.5	
Professional Degree	10.5	13.5	11	
Household Income				
<$10,000	2	1.5	2	0.047
$10,000-38,000	12	8	9.5	
$38,001-91,000	43	34	44.5	
$91,001-190,000	34	44	32.5	
>$190,000	9	12.5	11.5	
Using the Trails				
With Others	54.5	33	49	0.0001
Alone	45.5	67	51	
Using the Trails With …				
Spouse/Partner	42	30	50	0.0001
Exercise Partner	10	39	9.5	
Children	4.5	4	3	
Pets	8.5	1.5	0	
Coworkers	4	1.5	0.5	
Friends	20	9	19.5	
Relatives	4.5	1.5	4	
Walk/Run/Bike Club	0	6	0.5	
Mix of Above	6.5	7.5	13	
Time of the Day Using the Trails				
5–8 AM	10	13	10	0.1
8–11 Am	28	25	28	
11 AM – 2 PM	18	16	21.5	
2–6 PM	26	25	26.5	
After 6 PM	18	20	14	

Continuous variables were also compared and are presented in Table 2. Bikers spent a significantly longer time on trails per session than walkers and runners. They also showed a better mean score of mood than that the walkers and runners. The mean score of self-rated wellness and health in walkers was significantly lower than that in runners and bikers (Table 2). Mood and sleep results are shown in the Tables 3 and 4.

**Table 2:** Average (SD) values of continuous variables compared among the three types of physical activity on trails

Continuous Variables	Mean (SD)			
	Walkers	Runners	Bikers	*p* value
Years spent on using the trails	9.5 (6)	9 (5.5)	8.5 (5.5)	0.1
Miles performed for this the activity	3.5 (2)	5.5 (3)	15 (6)	0.001
Minutes spent on this activity per session	56 (34)	56 (31)	87 (50)	0.001
Days/week spent for this activity	4 (3)	3.5 (2.5)	3.5 (3)	0.6
Self-rated score of wellness & heath (0–10 when 10 is the best)	7.4 (1.5)	7.7 (1.3)	7.7 (1.4)	0.001
Diet score (0–14 when 14 is the worst)	4 (3)	4 (3)	3.5 (3)	0.07
Mood Score (0–35 when 35 is the worst)	7 (6)	7 (6)	6 (5.5)	0.004
Sleep Score (0–70 when 70 is the worst)	14 (10)	14 (10.5)	13 (9.5)	0.3

**Table 3:** Mood results of Gallup well-being index among all trail users

Occurrence of Symptom	Lack of Energy	Sadness	Anger	Physical Pain	Worry
Never	56%	64%	56%	49%	41%
Every Day	2%	3%	3%	11%	9%

**Table 4:** Sleep results of Mini-Sleep Questionnaire among all trail users

Symptom Rate	Difficulty Falling Asleep	Waking Up Too Early	Using Hypnotic Medica-tions	Falling Asleep During Day	Feel Tired Upon Waking	Snoring	Mid-sleep Awak-enings	Headache Upon Waking	Excessive Daytime Sleepiness	Excessive Movement During Sleep
Never	48%	43%	88%	64%	30%	57%	25%	81%	53%	69%
Every Day	3%	7%	4%	2%	8%	15%	26%	1%	3%	5%

The linear model was fitted for the self-rated wellness and health adjusting for important variables in walkers, runners and bikers, separately (Table 5). Age and mood were the only significant variables in all three models. Activity distance was also significant in runners and bikers. Sleep, smoking, and diet/education were significant in walkers, runners and bikers, respectively.

**Table 5:** Summary of estimates of three multivariable linear models built on self-rated wellness and health within each group of walkers, runners and bikers

Variables used to adjust three linear models	Estimates (β)		
	Walkers *n* = 302 *R*^2^ = 0.23	Runners *n* = 216 *R*^2^ = 0.32	Bikers *n* = 546 *R*^2^ = 0.32
Age	0.30*	0.40*	0.23*
Sex	0.03	0.10	0.05
Race	0.06	−0.09	0.06
Education	0.06	−0.06	−0.09*
Income	0.05	0.11	−0.02
Employment	−0.08	−0.15	− 0.10
Marital Status	−0.06	−0.13	−0.04
Diet	−0.06	−0.03	−0.05*
Smoking	−0.20	1.23*	0.42
Sleep	−0.03*	0.004	−0.007
Mood	−0.06*	−0.07*	−0.07*
Activity Distance	0.06	0.05	0.03*

Significant estimates are bolded and marked with star (*)

**Fig. 1:**
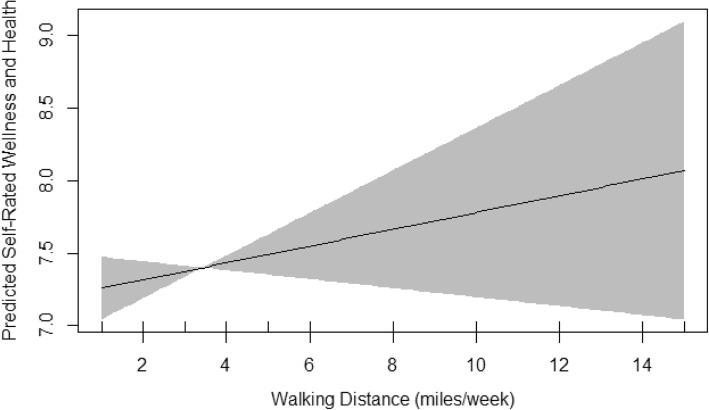
The Plot of Estimated Smoothing Spline Function of Walking Distance by Trail User With 95% Confidence Band for the GAM Model. The Response Variable Was Self-rated wellness and health

**Fig. 2:**
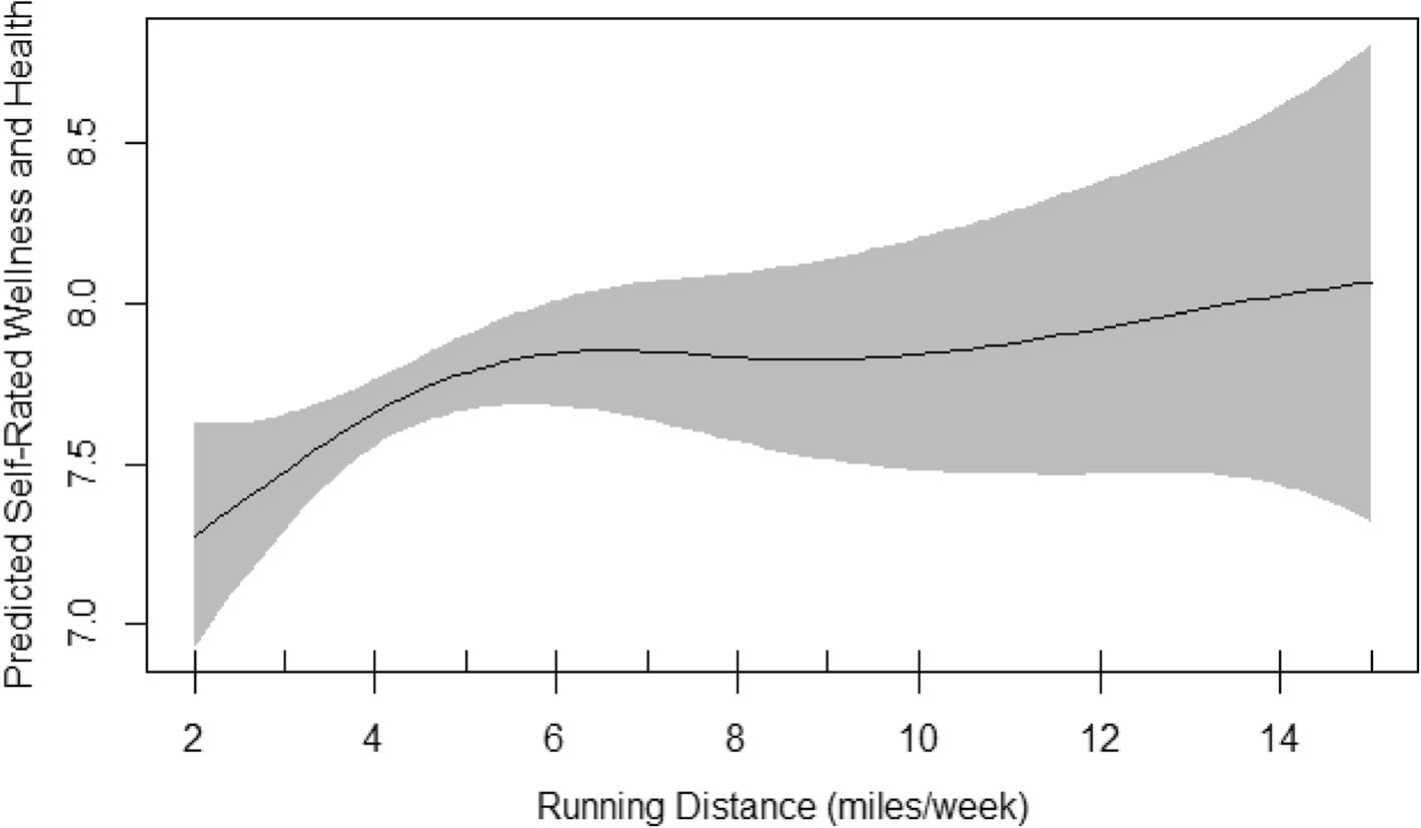
The Plot of Estimated Smoothing Spline Function of Running Distance by Trail User with 95% Confidence Band for the GAM Model. The Response Variable was Self-rated wellness and health

**Fig. 3:**
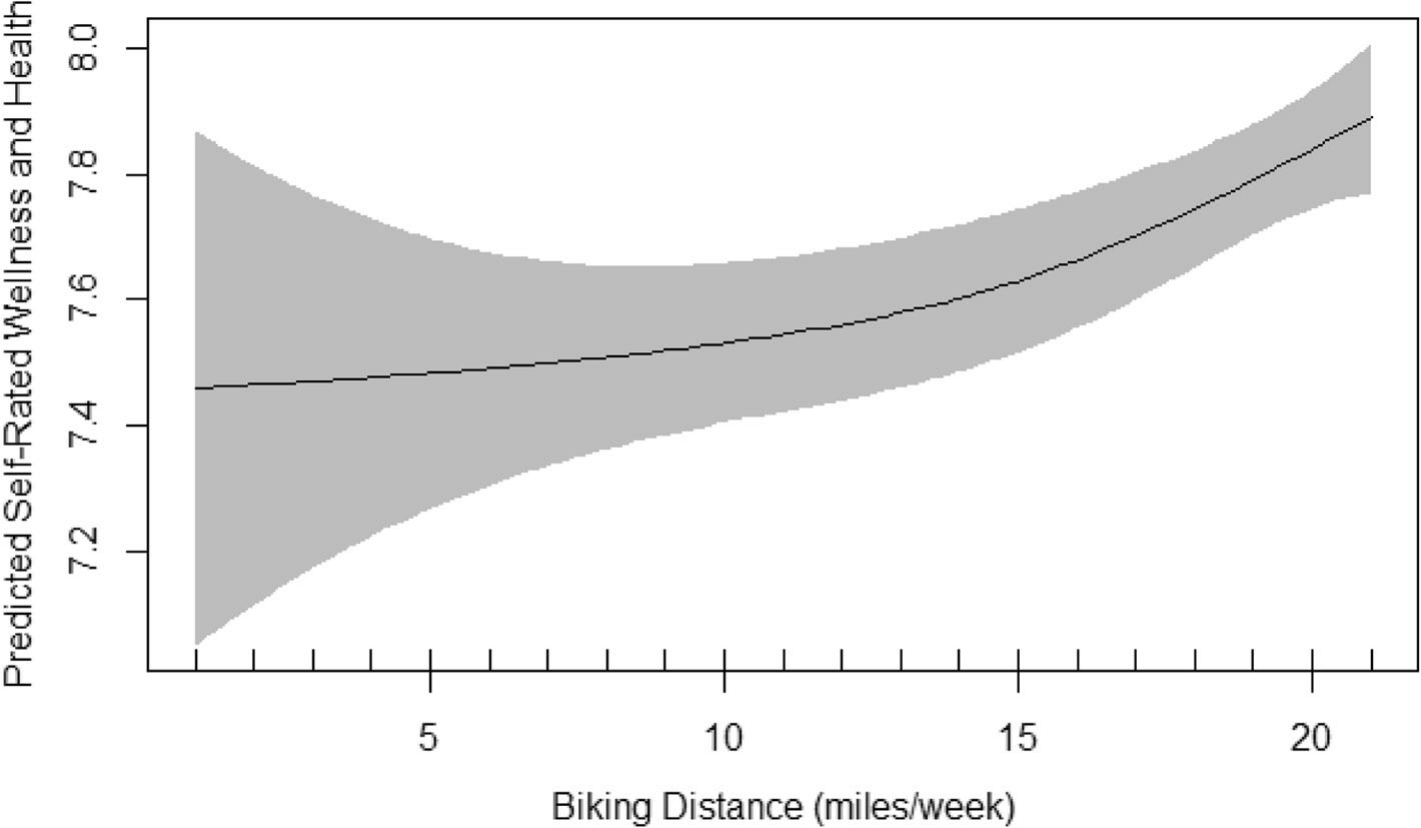
The Plot of Estimated Smoothing Spline Function of Biking Distance by Trail User with 95% Confidence Band for the GAM Model. The Response Variable was Self-rated wellness and health

Table 6 demonstrates the summary of GAM models including the estimates of significant coefficients, the main smoothing outputs and the important model characteristics including sample size (n) and R^2^. The normality of residuals and the equality of variances in GAM models were met. These assumptions were evaluated by basic checking plots and observing random symmetric scatter of the q-q plot around the plotted straight line, the normal distribution of the residuals’ histogram, the approximately constant variance of plot of residual vs. linear prediction, the positive relationship of the plot of residuals vs. fitted values with a good deal of scatter and finally, observing no evidence of outliers. Similar to linear models, age and mood were the only significant variables in all three models. Activity distance was also significant in bikers and almost significant in runners. Sleep, smoking, and diet were significant in walkers, runners and bikers, respectively. Only the model fitted on walkers showed an EDF of 1 which indicated a linear fit between walking distance and the self-rated wellness and health (Fig. 1). The EDF in the runners GAM model was 2.5 indicating a curved association between running distance and the self-rated wellness and health (Fig. 2). When runners were divided into two groups of < 6.5 miles running vs. ≥6.5 miles running, none of the demographic, health and well-being indices were different between the two groups except number of days per week that they ate less than four/five servings of fruits and vegetables; this was 3.1 vs. 2.4 days, respectively (*P* = 0.03). The EDF in the bikers GAM model was 1.7 indicating also curved association between biking distance and the self-rated wellness and health (Fig. 3).

**Table 6:** Summary of estimates of three multivariable GAM models built on self-rated wellness and health within each group of walkers, runners and bikers

Variables used to adjust three GAM models		Estimates (β)		
		Walkers *n* = 302 *R*^2^ = 0.23	Runners *n* = 216 *R*^2^ = 0.33	Bikers *n* = 546 *R*^2^ = 0.19
Age		0.30*	0.39*	0.24*
Sex		0.03	0.13	0.06
Race		0.06	−0.09	0.06
Education		0.05	−0.07	−0.09
Income		0.05	0.10	−0.02
Employment		−0.08	−0.15	−0.10
Marital Status		−0.06	− 0.14	−0.03
Diet		−0.06	−0.02	−0.05*
Smoking		−0.20	1.18*	0.44
Sleep		−0.03*	0.004	−0.007
Mood		−0.06*	−0.07*	−0.07*
Activity Distance	EDF	1	2.54	1.7*
	*P* value	0.19	0.07	**0.008**

Significant estimates are bolded and marked with star (*)

Final smoothing plots for walking, running and biking distance to predict the self-rated wellness and health are presented in Figs. 1, 2 and 3, respectively. The plots included the estimated penalized smoothing spline function with the linear effect subtracted out. Each plot also included a 95% confidence band for the whole curve/line. Figure 1 shows a constant linear association of walking distance and the self-rated wellness and health. The plots in Figs. 2 and 3 are compatible with the smoothness test results and confirmed graphically nonparametric (smooth) relationship of running/biking with the outcome, the self-rated wellness and health. The plot in runners (Fig. 2) indicated that running up to 6.5 miles per week was associated with a linear and sharp increase in self-rated wellness and health whereas running between 6.5 to 10 miles per week was not associated with any significant changes in self-rated wellness and health. Running more than 10 miles per week was again associated with linear but milder increase in self-rated wellness and health than running less than 6 miles per week. The plot in bikers (Fig. 3) indicated that biking more than 14 miles per week was significantly associated with steeper rise in self-rated wellness and health than biking less than 12–13 miles per week.

## Discussion

This was the first study that evaluated the semiparametric association of the trail activity distance and self-rated wellness and health. In two out of three GAM models, EDF was > 1 indicating the smooth (curved) association of running/biking distance and self-rated wellness and health. It means assuming linearity for the amount of running and biking is not appropriate for studying their association with outcome, self-rated wellness and health. The current study showed that the higher the walking distance, the higher the self-rated wellness and health. Also, up to 6.5 miles per week, the higher the running distance the sharper the increase in self-rated wellness and health. A similar association was observed for running more than 10 miles per week. The reason for the dip in health and wellness between 6.5 and 10 miles per week for runners is unclear. The reason could be their lower fitness level compared to more dedicated runners who run more than 10 miles per week or other hidden differences such as diet pattern that briefly explained above in Results section. Further qualitative studies of this finding are warranted. For biking, the results were different. The higher the biking distance after the first 14 miles per week, the sharper the rise in self-rated wellness and health. The GAM models in the current study was able to also roughly predict the self-rated wellness and health of Indiana trail users having their age, sex, race and other characteristics employed in building the models. The significant relationship between self-rated health and physical activities has been shown in several cross-sectional and cohort studies in Sweden [20–22], Greece [23], Spain [24], EU [25, 26], Syria [27], Korea [28], China [29], and Taiwan [30]. Given the wide heterogeneity of these international studies in terms of research methodology, population and sample size, an associated systematic review seems necessary to better estimate the magnitude of association. The current study was the first study that quantitatively showed the curved pattern of the relationship between self-rated wellness and health and type of physical activity among trail users.

Overall, employed educated married middle-aged women had the highest prevalence of walking among the walkers in the current study. Employed educated married young/middle-aged men had the highest prevalence of running among the runners. Similarly, employed educated married middle-aged men had the highest prevalence of biking among the bikers. Almost parallel findings were shown by other studies. For instance, employed educated middle-aged women had the highest prevalence of regular walking in Missouri [7]. Employed educated married middle-aged men also reported the highest prevalence of recreational biking in Australia [31].

Since the sample of rural and urban trails were selected from all over the Indiana state, the state’s overall demographic information is useful for the comparison with the demographics in the current study. The study results further illuminated health equity as an issue related to the use of trails. More specifically, the study found that more than 65% of trail users had a college education or advanced degree, 88% had a household income over $38,000 annually, and were predominantly white as shown in Table 1. The results, when compared to Indiana Statewide averages of $27,305 annual per capita income, 25.3% college degree or higher, and 85.1% white ethnicity [32] suggest that trail users were on the whole wealthier, more educated, and white compared to the rest of Indiana. This may highlight a potential health equity concern and the importance of education in promoting physical activity in future public health studies and interventions.

Acknowledging the significance of proximity and access to parks and related facilities such as trails can lower risk of cardiovascular disease, obesity, mental health, and other related health issues [33]. The current study did not identify trail user proximity to trail heads or park like areas. A growing body of research around access to natural areas, trees, and its linkage to increased physical activity with resulting health benefits shows improved physical health, socialization, and stress reduction as some of the benefits along with those associated with investment in underserved communities [4, 34]. These nature-based features are generally reflected in trail location and topography in many of the trail areas where participants are surveyed. Finally, important findings on demographics and trail use in the Santa Fe, NM region showed significant differences in trail use between Hispanics and non-Hispanics (45 and 85% respectively), further amplified by proximity to trails and safe infrastructure with 56% of non-Hispanic trail users having improved access to trails within 15 min of a trail in comparison to 31% of Hispanic trail users [35]. As the study was not specifically designed to measure nature access or inequitable access and proximity to trails, adapting future trail studies to include these issues is an important next step for further work on trail impacts to health.

Given the significant risk of obesity and depression among individuals with low physical activity and with rates of obesity and depression on the rise, the findings of this study will provide some rationale for the likelihood of improving wellness and health through various distances of trail activities and shows the necessity of building more trails throughout the country.

The current study contains several limitations. The cross-sectional design did not allow establishment of a causal relationship between the type of activity and self-rated wellness and health. Lack of information on the linkage between nature, physical activity, and access to trails was another limitation in the study. Diet was roughly controlled by asking two questions about fast food consumption and fruits/vegetables consumption. Obviously, there are other important elements in evaluating diet that were not measured. Volunteers were scheduled to recruit survey participants during specific times, but actual volunteer participation and effectiveness likely varied.

The strength of the current study was the application of GAM in finding the nonlinear curved-shape association of running/biking distance and self-rated wellness and health. Furthermore, the researchers tried to minimize the recall bias on seasonal variations by evaluating the trail users during all four seasons. Longitudinal studies could improve the reliability of study results. In addition, the relationship of self-rated wellness and health and trail physical activity was evaluated after controlling for all recognized confounders such as SES and mood [36], smoking [37], diet [17, 38], and sleep [39–42].

## Conclusions

The current study revealed the characteristics of population who currently use the trails in the state of Indiana and the patterns of their physical activities in trails. Employed educated married middle-aged people had the highest prevalence of walking, running and biking. It also demonstrated a linear association between walking and self-rated wellness and health; the higher the walking, the higher self-rated wellness and health. A similar association was observed for running up to 6.5 miles per week or biking > 14 miles per week. Future investigations could also assess the equity in access and the proximity to trails in follow-up studies.

## Data Availability

Data are available upon request through corresponding author.

## Abbreviations

EDF: Effective degree of freedom
GAM: Generalized additive models
RTES: Recreation Trail Evaluation Survey
SES: Socioeconomic status
